# Collective Dynamics of Model Pili-Based Twitcher-Mode Bacilliforms

**DOI:** 10.1038/s41598-020-67212-1

**Published:** 2020-07-01

**Authors:** Andrew M. Nagel, Michael Greenberg, Tyler N. Shendruk, Hendrick W. de Haan

**Affiliations:** 1 University of Ontario Institute of Technology, Faculty of Science, 2000 Simcoe Street North, Oshawa, Ontario L1H 7K4 Canada; 2 Interdisciplinary Centre for Mathematical Modelling and Department of Mathematical Sciences, Loughborough University, Loughborough, Leicestershire LE11 3TU UK; 3 School of Physics and Astronomy, The University of Edinburgh, Peter Guthrie Tait Road, Edinburgh, EH9 3FD UK

**Keywords:** Computational science, Computational biophysics

## Abstract

*Pseudomonas aeruginosa*, like many bacilliforms, are not limited only to swimming motility but rather possess many motility strategies. In particular, twitching-mode motility employs hair-like pili to transverse moist surfaces with a jittery irregular crawl. Twitching motility plays a critical role in redistributing cells on surfaces prior to and during colony formation. We combine molecular dynamics and rule-based simulations to study twitching-mode motility of model bacilliforms and show that there is a critical surface coverage fraction at which collective effects arise. Our simulations demonstrate dynamic clustering of twitcher-type bacteria with polydomains of local alignment that exhibit spontaneous correlated motions, similar to rafts in many bacterial communities.

## Introduction

Active matter possesses the potential to bridge between physics and biology. Like living systems, manufactured active systems maintain far-from-equilibrium states by autonomously drawing energy from the surroundings to fuel non-thermal processes. Furthermore, active systems exhibit many of the characteristic traits of biological materials, such as spontaneous motion, self-organization and complex spatio-temporal dynamics. Communities of model bacteria, such as *Pseudomonas aeruginosa*, are excellent biological examples of out-of-equilibrium systems. These relatively simple living systems serve as a biophysical study of active matter in which collectivity arising from bio-mechanical action can perform essential biological roles.

Theories and simulations have approached such bacterial systems by simplifying or omitting all but the most essential, lowest-order physical traits of these microbes, as well as biological complexities. From the very first considerations of active matter, self-propulsion and local alignment were identified as the fundamental components necessary for collective dynamics to emerge from active particles^1,2^. Simulations of self-propelled rods and their continuum limit of active nematics have been particularly important to the field^3–14^, as recently reviewed in ref. ^15^. However, the universality of behaviors exhibited by active systems is still a matter of debate^16,17^ and it cannot simply be taken for granted that the collective dynamics of Vicsek boids^1^, active Brownian particles^18–20^ or self-propelled rods are directly inherited by microbial motility strategies. Indeed it is known that what might appear to be higher-order details can qualitatively alter the large-scale dynamics. For example, while self-propelled rods and other active colloids commonly exhibit pronounced clustering^21^, which can be explained by motility-induced phase separation or other theoretical approaches^22–24^, swimming microbes can behave as homogeneous fluids on the scales of mesoscale active turbulence^25^, with simulations suggesting that the details of hydrodynamic interactions are essential for differentiating these large-scale swimmer properties^20,26–28^. Various modes of swimming motility, including but not limited to pushing, pulling, squirming and undulating, as well as their microscopic details, have been extensively considered^29–31^. However, swimming is only one of many motility mechanisms employed by *P. aeruginosa* and other motile microbes^32^. Other motility modes employed by *P. aeruginosa* alone include swarming^33^, hyperswarming^34^, sliding^35^, walking^36^, slingshot^37^, and twitching^38^, not to mention the migration modes of many eukaryotic cells^39^. While these motility strategies have received less attention than swimming modes, each has the potential to introduce seemingly microscopic details from which emerge distinctive collectivity.

Twitching motility plays a particularly critical role in redistributing cells on surfaces prior to colony and subsequent biofilm formation^38,40–42^, as well as impacting final biofilm morphology^43,44^ and compositional structure^45–47^. Twitching motility is a flagella-independent form of translocation over moist surfaces, commonly studied using motility plate assays of 1% agar^48^. Twitching motility relies on type-IV pili^49^, which are filamentous appendages common to many gram-negative, and some gram-positive bacteria^47,50^. Through an active cycle of pili extension, anchoring and retraction^51,52^, *P. aeruginosa* and other bacilliforms can jerkily crawl over surfaces. This twitching activity enables rapid dissemination and invasion, while it is simultaneously capable of bringing cells together into locally crowded configurations. As simulations of swimming-mode motility have demonstrated that the details of swimming produce essential consequences not seen in simple self-propelled rods^53^, so too it is constructive to simulate and quantify the collective dynamics of model twitcher-mode bacteria and to quantify any distinctions between dynamics in the low and high density regimes.

**Figure 1:**
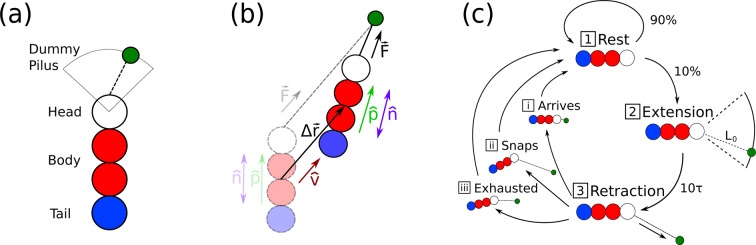
**Schematics describing the twitcher model.** **(a)** Single twitcher discretized into four Langevin spheres. A dummy pilus extends from the head particle and is affixed to the surface stochastically within a cone $[-\pi \mathrm{/4,}\,\pi \mathrm{/4}]$, while it applies a constant retraction force on the head. **(b)** The motion of a single twitcher described by its pilus force $\overrightarrow{F}$, the center of mass displacement $\Delta \overrightarrow{r}$, the direction of motion $\hat{v}$, polar orientation $\hat{p}$, and nematic alignment $\hat{n}$. **(c)** The motility cycle of a single twitcher. The twitcher is non-motile in the rest (1) and extension phases (2) but pulls itself forward during the retraction phase (3). A resting twitcher has a $\mathrm{90 \% }$ probability per time step $\tau$ to continue resting. The extension of the pilus to an adhesion point a distance ${L}_{0}$ from the head takes $10\tau$. The retraction phase continues until: (i) the head arrives at the adhesion point, (ii) the head is pushed too far from the dummy pilus point causing the pilus to snap, (iii) the adhesion is exhausted after a maximum adhesion time ${t}_{{\rm{M}}}$.

We present the results of a coarse-grained model that accounts for biologically relevant twitching motility of rod-like bacilliforms fixed to a planar surface. Motivated by twitcher-mode bacterial dynamics, this model goes beyond traditional self-propelled rods, which typically consist of a persistent force aligned along the body of each rod subject to continuous noise distributions^15^. As shown schematically in Fig. 1, the mechanics of twitching are modelled dissimilarly from traditional self-propelled rods. In this study, each bacilliform twitcher stochastically obeys a twitching cycle of rest, pili extension and active retraction. Thus, at any instant, our simulations contain a mixture of active and passive bacteria, which allows us to observe the effect of the passive bacteria on the emergence of collective motion and also how passive substances can be swept along with active neighbors. Further differentiating our model from studies of traditional self-propelled rods, model twitchers employ a dummy pilus (Fig. 1a), which pulls the bacteria body towards a fixed adhesion point on the substrate. This dummy-pilus scheme means that the propulsive bearing, direction of motion and orientation can each be markedly different. Thus, to study the collective motion of twitching mode bacteria, we have developed a distinct model. Nonetheless, our model neglects further biological complications, such as multiple motility modes^40,42,48^, reproduction^54^, biosurfactants^55^, bacteria-secreted polymeric trails^56^ and nutrient competition. Incorporation of these effects is left to future work.

To the best of our knowledge, this report is the first numerical study of the collective effects that can arise from twitching mode motility and our simulations explicitly demonstrate that collective motion can arise from purely physical mechanisms. That is, with a sufficiently high coverage fraction, rod-like twitchers nematically align through excluded-volume interactions and form dynamic clusters that exhibit correlated motion. However, we also make clear that the emergent collectivity is not immediately apparent through a transition to flocking or swarming, nor through a qualitative change in the mean squared displacements. Rather, we quantify the dynamics through changes to the non-Gaussian parameter, relative diffusivity and decorrelation lengths, which together constitute a suite of statistical tools readily available to experimentalists studying the collective dynamics of twitching bacteria, such as *P. aeruginosa*.

## Results

**Figure 2:**
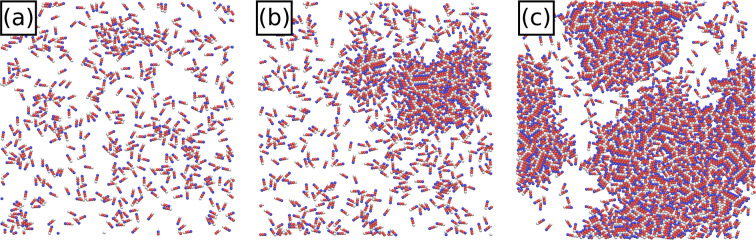
**Simulation snapshots.** **(a)** Surface coverage $\phi =0.19$ (Supplemental Movie 3). **(b)** Near the critical surface coverage $\phi =0.3\approx {\phi }^{\ast }$ (Supplemental Movie 4). **(c)** High surface coverage $\phi =0.57$, exhibiting coexistence of a locally dilute phase and a dense phase with non-homogeneous polydomains of orientational ordering (Supplemental Movie 5).

**Figure 3:**
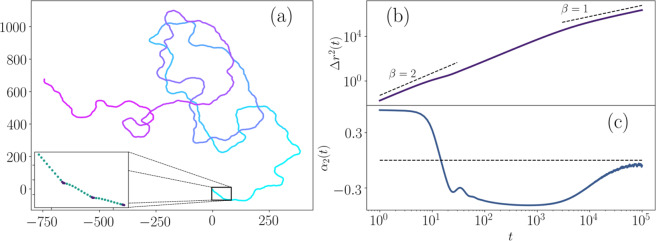
**Solitary twitcher dynamics.** **(a)** Example trajectory. (**Inset**) Short time showing resting/extension and retraction phases. **(b)** Mean squared displacement $\Delta {r}^{2}\sim {t}^{\beta }$, with propulsive behavior ($\beta \approx 2$) at short/intermediate times and diffusive behavior ($\beta \approx 1$) at long times. **(c)** Non-Gaussian parameter ${\alpha }_{2}(t)$, which is zero for Gaussian statistics, $\mathrm{ < 0}$ when there are fewer large displacements than a normal distribution with the same second moment, and $\mathrm{ > 0}$ when there are more.

Our coarse-grained simulations of bacilliform microbes treat each individual twitcher as a stiff chain of four spheres with dynamics obeying Langevin equations of motion^57,58^, with a non-integrated dummy particle representing the action of bacterial pili (Fig. 1a). Excluded-volume, finite-extension connectivity and rigidity are each accounted for via potentials as described in detail in the Methods Section. All quantities are expressed in terms of simulation units with length in terms of twitcher sphere size $\sigma$, mass in sphere mass $m$, energy in Lennard-Jones well-depth $\varepsilon$ and unit time $\tau =\sqrt{m{\sigma }^{2}/\varepsilon }$. Twitching motility is modeled via the pilus particle, which actively pulls each individual twitcher forward (Fig. 1b) and obeys a stochastic rule-based cycle composed of three phases (Fig. 1c):

1. The first phase is the **rest** phase. Resting twitchers do not do not undergo self-induced movement, only passively respond to external forces and have a 10% chance per $\tau$ of stochastically transitioning to the next phase (Fig. 1c-1).
2. The next phase is the **extension** phase, in which the dummy pilus extends over a set period of $10\tau$ then adheres to the surface a distance ${L}_{0}=2.4$ away from the head particle with a random angle between $-\pi \mathrm{/4}$ and $\pi \mathrm{/4}$ (Fig. 1c-2).
3. The **retraction** phase is the period in which the twitcher is actively motile (Fig. 1c-3). The twitcher’s head is pulled towards its fixed pilus adhesion point with a force $\overrightarrow{F}$ of constant magnitude to model the average force exerted by multiple pili^59^. The retraction phase ends when one of three conditions are met:
  (i) The twitcher **arrives** at its pilus adhesion point, which is achieved if the distance between the head and the pili adhesion point ${r}_{\gamma }$ is less than the cutoff ${L}_{{\rm{R}}}=0.2$ (Fig. 1c-3.i).
  (ii) The pilus adhesion **snaps** because the head is pushed further from the adhesion point than the cutoff ${L}_{{\rm{S}}}=3$ (Fig. 1c-3.ii).
  (iii) The adhesion is **exhausted** if the retraction phase persists for more than ${t}_{{\rm{M}}}=70\tau$ (Fig. 1c-3.iii).

Once any of these occur, the twitcher returns to the rest phase and the cycle repeats.

The instantaneous state of the ${\gamma }^{{\rm{th}}}$ twitcher is quantified by its center of mass position ${\overrightarrow{x}}_{\gamma }(t)$, velocity ${\overrightarrow{v}}_{\gamma }(t)$ and orientation. The velocity is defined as the displacement vector $\Delta \overrightarrow{r}$ per time step (Fig. 1b), along with associated speed ${v}_{\gamma }(t)=|{\overrightarrow{v}}_{\gamma }|$ and direction of motion ${\hat{v}}_{\gamma }(t)={\overrightarrow{v}}_{\gamma }/{v}_{\gamma }$. The direction of motion does not necessarily align with the retraction force $\overrightarrow{F}$, nor the orientation (Fig. 1b). We consider both the polar orientation ${\hat{p}}_{\gamma }(t)$, the unit vector pointing from tail to head, and the rod-like nematic alignment, for which ${\hat{n}}_{\gamma }(t)$ and $-{\hat{n}}_{\gamma }(t)$ are equivalent. Twitchers interact with one another through excluded-volume repulsion and we define the coverage fraction to be the area of $N$ twitchers normalized by the 2D simulation box size. We simulate a wide variety of coverage fractions, from a solitary twitcher ($N=1$ and $\phi =4\times {10}^{-4}$) to $N=2000$ ($\phi =0.76$). Supplemental Movies 1–6 illustrate the simulation results for surface coverages $\phi =\{4\times {10}^{-4},0.04,0.19,0.3,0.38,0.57\}$ respectively, snapshots from which are shown in Fig. 2. Further details are available in the Methods Section.

## Solitary Twitcher

In the absence of interactions with other twitchers, the dynamics of a solitary twitcher are controlled entirely by the motility cycle (Section Motility Cycle). Example trajectories appear diffusive on long times (Fig. 3a; Supplemental Movie 1), though closer inspection of shorter periods demonstrates the rest/extension and active retraction phases, as well as correlated motion across multiple resting phases (Fig. 3a; inset). The consequences of these phases can be characterized by calculating the mean square displacement (MSD)

1 $$\Delta {r}^{2}(t)\equiv \langle {|{\overrightarrow{x}}_{\gamma }\mathrm{(0)}-{\overrightarrow{x}}_{\gamma }(t)|}^{2}\rangle $$

as a function of lag time $t$ from any initial time (Fig. 3b). MSD is a natural measurement for situations involving randomness, in which case the average of the displacement $\Delta r(t)\equiv \langle {\overrightarrow{x}}_{\gamma }\mathrm{(0)}-{\overrightarrow{x}}_{\gamma }(t)\rangle$ is often zero. As a measure of the width of the distribution of step sizes for each lag time, MSD measures the extent of the random motion. The lag time is simply the time interval from the arbitrarily chosen starting point. The manner in which MSD increases as a function of lag time can help us to understand the nature of twitchers’ motion. At different lags, we observe $\Delta {r}^{2}\sim {t}^{\beta }$, where the scaling $1\le \beta (t)\le 2$, with $\beta =1$ indicating diffusive dynamics and $\beta =2$ signaling propulsive motion. For short times $t\lesssim 10$, $\Delta {r}^{2}(t)$ scales as $\beta =2$, corresponding to active self-propelled motion of a single retraction phase dominating over the noise induced by the random pilus extension angle. From *t* ≈ 10–30, there is a shoulder in the MSD where $\Delta {r}^{2}(t)$ nearly saturates, illustrating the pauses in self-propelled motion during the rest and extension phases. In contrast to our model, isolated *P. aeruginosa* cells extend pili to variable lengths and retraction times are stochastic^60^, which would be expected to dampen the shoulder in our numerical model. After $t\gtrsim 30$, $\Delta {r}^{2}(t)$ again scales as $\beta \approx 2$, indicating correlated motion across multiple twitching jumps due to the model restricting pilus adhesion to a cone in front of the twitcher (see Fig. 1a). Around $t\gtrsim {10}^{3}$, the scaling transitions to $\beta \approx 1$, corresponding to diffusive dynamics over long lag times and indicating a random walk as expected from the random motion exhibited in Fig. 3a.

For such a rich motility cycle, the MSD does not exhibit compelling qualities, only hinting at the underlying dynamics as described above. While the MSD tells us the width of the distribution of step sizes for each lag time, it cannot tell us more. Indeed there has been a growing appreciation in the soft condensed matter community that the MSD can easily be over interpreted^61–66^ (as recently reviewed in ref. ^67^) and this issue requires even greater care in biologically complex systems, such as ensembles of twitching *P. aeruginosa*. To learn more, we need to consider more subtle aspects of the displacements and we turn to higher order moments of the the displacement distribution. The extent to which the dynamics deviate from the Gaussian distribution, which would lead to diffusive motion, can be measured by a non-Gaussian parameter (NGP)^68–70^

2 $${\alpha }_{2}(t)=\frac{d}{d+2}\frac{\Delta {r}^{4}}{{|\Delta {r}^{2}|}^{2}}-\mathrm{1,}$$

where the dimension $d=2$ since the twitchers are confined to a plane and $\Delta {r}^{4}=\langle {|{\overrightarrow{x}}_{\gamma }\mathrm{(0)}-{\overrightarrow{x}}_{\gamma }(t)|}^{4}\rangle$. While the MSD gives the second order moment of the displacement distribution, NGP gives the fourth moment and so expresses information about the motion that is not generally encoded in the MSD ($\Delta {r}^{4}\ne {|\Delta {r}^{2}|}^{2}$ in general). However, in the particular case of a Gaussian distribution all higher order even moments are functions of the MSD; particularly, the fourth moment of a normal distribution is $\Delta {r}^{4}=(d+\mathrm{2)}{|\Delta {r}^{2}|}^{2}/d$, which would give ${\alpha }_{2}(t)=0$. Thus, NGP communicates the extent to which the displacement distribution differs from normal. When ${\alpha }_{2}(t) < 0$ the displacement distribution is said to be platykurtic, meaning there are fewer large step sizes than would be produced by a normal distribution with the same second moment. When ${\alpha }_{2}(t) > 0$ the distribution is leptokurtic, indicating that the tails of the distribution are longer than normal.

**Figure 4:**
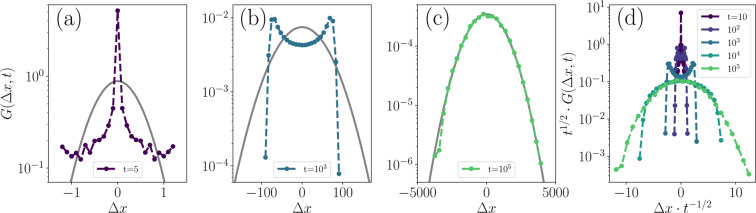
**Solitary twitcher step size distributions.** **(a–c)** Distribution $G(\Delta x,t)$ for various lag times $t$ and step sizes $\Delta x$ along either Cartesian axis. Grey curves denote reference Gaussian distributions with equivalent standard deviations to the respective step size distributions. **(d)** Step size distributions normalized to collapse diffusive curves.

The NGP much more clearly indicates the three regions that could be discerned from the MSD (Fig. 3c). Moreover, it reveals the dynamics at each of these time scales to be leptokurtic, platykurtic and normal, respectively. Additionally, to demonstrate these different regimes explicitly, the distribution of twitcher displacements $G(\Delta x,t)$ is calculated and compared to Gaussian distributions with the same standard deviation. These distributions, which are sometimes referred to as van Hove self-correlation functions^65^, are shown in Fig. 4 for several times.

Firstly, ${\alpha }_{2}(t)$ in Fig. 3c approaches zero at long times, indicating Gaussian dynamics just as the MSD indicated diffusive behavior. This is verified in Fig. 4c where $G(\Delta x,t={10}^{5})$ closely matches the equivalent Gaussian curve. Next, at the shortest lag times in Fig. 3c, ${\alpha }_{2}(t)$ approaches a positive constant of $\sim 0.55$ because the twitchers are likely to be found in the motile retraction phase with large propulsive displacements. This leptokurtic behaviour is shown explicitly in Fig. 3a where the tails of the $G(\Delta x,t=\mathrm{5)}$ distribution are much longer than those of the Gaussian. The sharp peak at zero displacement reflects the non-motile rest phases. Finally, between these limits, ${\alpha }_{2}(t)$ is platykurtic and approaches the lower bound of $-\mathrm{2/(}d+\mathrm{2)}$^71^, which reflects the sequential resting phases that shorten the tails of the displacement distribution in comparison to a random walk. Figure 4b displays the distribution of step sizes at $t={10}^{3}$ and the platykurtic nature is evident from the sharply truncated tails of $G(\Delta x,t={10}^{3})$ compared to the Gaussian. This, coupled with the sharp shoulders, indicate the greater likelihood of traveling in a correlated manner but then abruptly pausing to rest with only a vanishingly small probability of traversing any further. Recall that $\beta \approx 2$ for both the leptokurtic and platykurtic regimes in the MSD, and so the qualitative difference in dynamics could only be quantified by considering the NGP.

Figure 4d displays the step size distributions at 5 different times. The $\Delta x$ values are scaled by ${t}^{-\mathrm{1/2}}$ and the distributions are normalized by ${t}^{\mathrm{1/2}}$ such that curves corresponding to pure diffusion would collapse. This allows examination of the evolution of $G(\Delta x,t)$ across disparate time scales. The decay of the sharp peak at $\Delta x=0$ at short times, the emergence of sharp shoulders and cut tails at intermediate times, and the convergence towards a universal curve indicating diffusion at long times are all evident.

## Collective Twitcher Dynamics

### Individual dynamics of constituent twitchers

**Figure 5:**
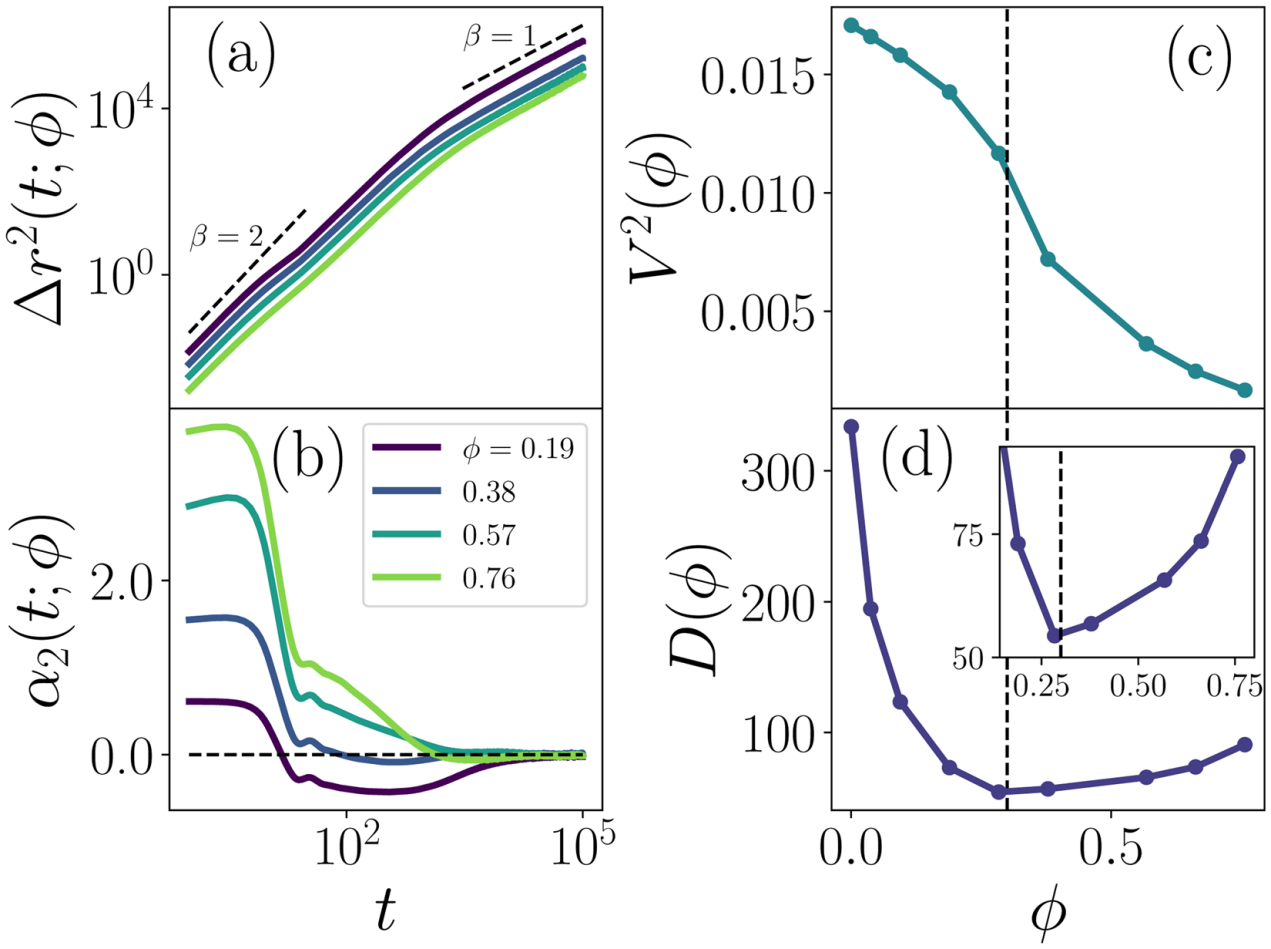
**Collective dynamics of twitcher systems of different coverage fractions** ***ϕ***. **(a)** Mean squared displacement $\Delta {r}^{2}(t;\phi )$. **(b)** Non-Gaussian parameter ${\alpha }_{2}(t;\phi )$. **(c)** Short-time mean squared velocity ${V}^{2}(\phi )=\Delta {r}^{2}(\tau ;\phi )/{\tau }^{2}$. The vertical dashed line marks the critical coverage ${\phi }^{\ast }$. **(d)** Long-time relative diffusion $D(\phi )={\mathscr{D}}(\phi )/\tau {V}^{2}(\phi )$, where ${\mathscr{D}}(\phi )$ is the diffusion coefficients as measured from the MSD for $t > {10}^{4}$. **(Inset)** High coverage regime.

To assess pre-colony collective dynamics as a function of surface coverage, we simulate ensembles of twitchers. At low coverage ($\phi =0.19$ curve in Fig. 5a; Supplemental Movies 2–3), the mean squared displacement retains the qualities observed in the solitary twitcher case: the short-time active self-propulsion with scaling *β* = 2; shoulder near *t* ≈ 10–30 due to the non-motile rest phases; correlated motion across multiple twitching jumps (intermediate times) with *β* ≈ 2; and random-walk dynamics at long times with *β* = 1 (Fig. 5a). In fact, as the coverage fraction further increases ($\phi =0.57,0.76$ curves), the MSD curves remain qualitatively similar. The shoulder in $\Delta {r}^{2}(t;\phi )$ in the vicinity of *t* ≈ 10–30 becomes less pronounced; however, the scaling $\beta$ for short, intermediate and long times is essentially unaffected. However, increasing *ϕ* does cause two limiting changes to the twitcher MSD:

1. At short times, the MSD curves shift down as *ϕ* increases (Fig. 5a). In this short-time regime, $\Delta {r}^{2}(t;\phi )\sim {t}^{2}$. Thus, we define an effective short-time mean squared velocity (MSV) by ${V}^{2}(\phi )=\Delta {r}^{2}(\tau )/{\tau }^{2}$ (Fig. 5c). Starting from low coverage fractions, the MSV decreases relatively weakly with increasing *ϕ* because the twitchers are well separated and seldom collide. The MSV decreases because collisions become more likely, generally slowing active twitcher motility.
2. At long times, the MSD curves are diffusive and the $d=2$ dimensional diffusion coefficient can be extracted by fitting $\mathop{lim}\limits_{t\to {\rm{\infty }}}\Delta {r}^{2}(t;\phi )=2d{\mathscr{D}}t$. However, the reduction of the short-time ${V}^{2}(\phi )$ has already slowed the dynamics, effectively acting as an increased viscosity at long times causing the effective diffusion coefficient ${\mathscr{D}}$ to decrease with increasing *ϕ*. To normalize, we consider the dimensionless relative diffusivity $D(\phi )={\mathscr{D}}(\phi )/\tau {V}^{2}(\phi )$ (Fig. 5d). The relative diffusivity is non-monotonic with its minimum corresponding to the same surface coverage as the inflection point in ${V}^{2}$.

By considering the limiting character of the MSDs we are able to extract some subtle differences in the collective behavior that is not immediately apparent. However, while the MSD curves remain qualitatively similar at all surface coverages, the non-Gaussian parameters reveal a qualitatively distinct change to the collective dynamics (Fig. 5b). At low coverage, the NGP manifests the same three regimes as the solitary case (Fig. 3c) but comparing the $\phi =0.19$ (Supplemental Movie 3) and $\phi =0.38$ (Supplemental Movie 5) curves in Fig. 5b, the transition to ${\alpha }_{2}(t;\phi )\approx 0$ diffusion from the negative platykurtic plateau occurs at earlier times as *ϕ* increases, illustrating the loss of the distribution’s large displacement tails. This shift is due to collisions between twitchers randomizing the correlated motion between retraction phases earlier than in the solitary limit. As the surface coverage increases, ${\alpha }_{2}(t;\phi )$ loses the negative plateau altogether, becoming leptokurtic at all but the longest lag times, *i.e*. revealing the distribution has longer tails than expected for a Gaussian despite the rest phase. This is accompanied by a change in the short-time limit of ${\alpha }_{2}(t;\phi )$: sparse surface coverage ($\phi =0.19$ curve) exhibits a constant $\mathop{lim}\limits_{t\to 0}{\alpha }_{2}(t;\phi )\approx 0.5$; however, it rises substantially. This implies that larger displacements than expected by a normal distribution become far more common at both short and intermediate time scales.

This is a strong indication of collective and coherent motion at intermediate time scales suggesting that even rest-phase twitchers are typically moving due to interactions with retraction-phase neighboring twitchers, with more frequent large step sizes than expected for diffusive motion. This indicates a qualitative change in twitcher behavior, which can be understood as the transition from distinct collision events at low coverage ($\phi  < {\phi }^{\ast }$) to continuous interactions at high coverage ($\phi  > {\phi }^{\ast }$). This roughly suggests

3 $${\phi }^{\ast }=\frac{{A}_{{\rm{twitch}}}}{\pi {({L}_{{\rm{body}}}\mathrm{/2)}}^{2}}\approx 0.3$$

to be the point at which the mean area per twitcher equals the characteristic rotational area occupied by each twitcher and above which ${\alpha }_{2}(t;\phi )\ge 0$ at all lag times. The importance of ϕ^*^ on the dynamics is also discernible from the MSV (Fig. 5c) and relative diffusivity (Fig. 5d). While the decrease in MSV is monotonic, there is an inflection point at $\phi \approx {\phi }^{\ast }$. Similarly, systems with low coverage fractions have the largest $D(\phi )$, as twitchers seldom obstruct each other’s diffusive motion, which remains the case until ${\phi }^{\ast }$ (Supplemental Movie 4), at which point the relative diffusivity $D(\phi )$ is a minimum (Fig. 5d). Beyond ${\phi }^{\ast }$ (Supplemental Movies 5–6), $D(\phi )$ increases, further demonstrating the collective motion that emerges at high coverage.

**Figure 6:**
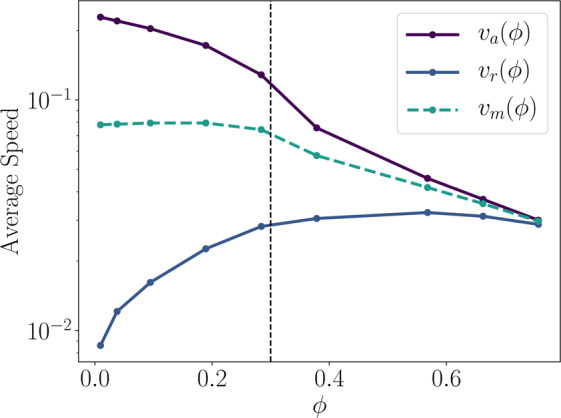
**Average speed of twitchers.** The total weighted average speed ${v}_{{\rm{m}}}(\phi )$ is separated into the contributions from twitchers in their resting/extending state ${v}_{{\rm{r}}}(\phi )$ and their active retraction state ${v}_{{\rm{a}}}(\phi )$. Critical coverage ϕ^*^ denoted with dotted vertical line.

To further understand this collectivity, we consider the average speed ${v}_{{\rm{m}}}(\phi )$ (Fig. 6; green dashed). We show the separated contributions due to twitchers in their actively self-motile retraction phase ${v}_{{\rm{a}}}(\phi )$ (Fig. 6; purple) and their resting/extending non-motile phases ${v}_{{\rm{r}}}(\phi )$ (Fig. 6; blue). The mean ${v}_{{\rm{m}}}(\phi )$ is constant for low coverages and only decreases substantially once $\phi  > {\phi }^{\ast }$. On the other hand, ${v}_{{\rm{a}}}(\phi )$ decreases in both regimes. In the intermediate $\phi \approx {\phi }^{\ast }$ regime, we see that slight increases in $\phi$ result in large decreases in ${v}_{{\rm{a}}}(\phi )$. At this coverage, neighboring twitchers are hindering each others’ motion but are not recompensing significant speed through collective effects, as will occur at higher coverages.

Figure 6 demonstrates that even twitchers in the resting phase of the motility cycle are collectively advected as *ϕ* approaches the critical coverage. In fact, ϕ^*^ clearly marks the saturation of the increase in the speed of resting twitchers, a sharp decrease in the speed of active twitchers, and the beginning of the decrease in the mean speed. It is interesting to compare this to typical rod models where the rods experience steadfast self-propulsion and uniform orientational noise and thus are never inactive^15^. The critical coverage as calculated from Eq. 3 is a purely geometric argument; it does not consider what fraction of the matter covering the surface is active. This estimate of ϕ^*^ works well for both continuous self-propelled rods and the mix of active/passive twitchers studied here thus indicating that the emergence of collective motion is primarily dictated by excluded volume effects rather than energetic considerations.

**Figure 7:**
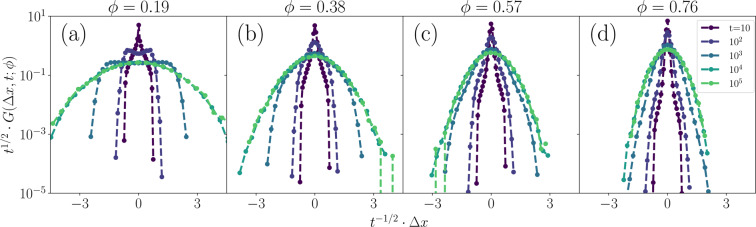
**Step size distributions.** Van Hove functions $G(\Delta x,t;\phi )$ with axes normalized to collapse diffusive dynamics. Panels a-d show step size distributions for various coverage fractions ($\phi =0.19,0.38,0.57,0.76$).

While ${v}_{{\rm{a}}}(\phi )$ decreases, ${v}_{{\rm{r}}}(\phi )$ rises with the frequency of collisions between twitchers. In fact, by the highest coverage fractions, there is sufficient collective motion for the rest/extension phase twitchers to be advected at the same average speed as the retracting twitchers (Fig. 6). These dynamics are explained by the step size distribution $G(\Delta x,t;\phi )$ (Fig. 7). Focusing on the $\phi =0.19$ subplot ($\phi  < {\phi }^{\ast }$), $G(\varDelta x,t;\varphi )$ is similar to the solitary twitcher limit shown in Fig. 3d. However, as the coverage surpasses ϕ^*^ in the remaining three subplots, the intermediate-time peaked shoulders become suppressed (Fig. 7b–d). This is because collisions make it both unlikely to remain in place during rests and unlikely to travel without obstruction for long periods. At intermediate *ϕ*, moderate lag times ($t={10}^{3}$ in Fig. 7(b,c)) begin to collapse on to the long-time diffusive distributions, which itself narrows with increasing *ϕ*. At the highest *ϕ* (Fig. 7d), the intermediate lag time $G(\Delta x,t;\phi )$ of $t={10}^{3}$ again transitions — now behaving like the short-time distributions.

The notion that resting twitchers do not impede the emergence of collective motion and can actually exhibit speeds comparable to that of active twitchers at large *ϕ* is in agreement with previous studies. It has been shown experimentally that not all twitching cells must be motile in order to exhibit collective effects^38^ and that polystyrene microspheres can be moved across surfaces by colonies of twitching *P. aeruginosa*^72^. Further, physical studies of active granular matter have demonstrated that collectivity can arise in systems consisting of few active agents surrounded by many passive particles^73^. The results shown in Figs. 6 and 7 demonstrate that this is true not only for non-motile tracers, species, or mutants, but rather is continually occurring for resting cells.

Returning to the step-size distributions at large *ϕ* results depicted in Fig. 7, the short-time center peak and long tails indicate that the majority of individual twitchers are caged by their neighbors but are able to collectively advect and so move larger distances than expected if they were behaving diffusively. These caging effects are particularly evident in the correlated motion of individual twitchers within the ensemble. To explore how persistent the direction of motion of individual twitchers is, we consider the spatial individual auto-correlation (IAC) function of the direction of motion ${\hat{v}}_{\gamma }$ of the ${\gamma }^{{\rm{th}}}$ twitcher along its own trajectory. The IAC is given by

4 $${\rho }_{\hat{v}}(\Delta r;\phi )=\langle {\hat{v}}_{\gamma }(0)\cdot {\hat{v}}_{\gamma }(\Delta r)\rangle ,$$

where $\Delta r$ is the distance travelled relative to an arbitrary starting point. When $\Delta r$ is small, no twitcher will have moved far nor changed direction and ${\hat{v}}_{\gamma }(0)$ and ${\hat{v}}_{\gamma }(\Delta r)$ will be very similar, such that $\langle {\hat{v}}_{\gamma }(0)\cdot {\hat{v}}_{\gamma }(\Delta r)\rangle \approx 1$. As each twitcher moves across the surface, $\Delta r$ increases and the correspondence between the direction of motion at the starting point and at $\Delta r$ is lost. In the limit of completely uncorrelated directions of motion, $\langle {\hat{v}}_{\gamma }(0)\cdot {\hat{v}}_{\gamma }(\Delta r)\rangle$ approaches zero. The IAC defined in Eq. 4 thus decays from $\approx 1$ to small values with increasing $\Delta r$ thus indicating how the direction of motion is randomized with increasing displacement. Note that the IAC is averaged over both initial times and the ensemble of twitchers. Similar auto-correlation functions have previously proven useful in assessing collective motion of swimming *Bacillus subtilis*^74–77^.

**Figure 8:**
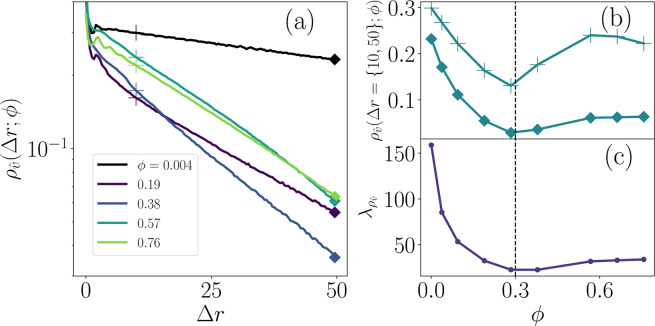
**Individual auto-correlation (IAC) within an ensemble.** **(a)** IAC function ${\rho }_{\hat{v}}(\Delta r;\phi )$ of the direction of motion of an individual twitcher. **(b)** ${\rho }_{\hat{v}}(\Delta r;\phi )$ for two values of distance traveled $\Delta r(t)=\{10,50\}$ as a function surface coverage *ϕ*. Markers denote $\Delta r=10$ (+) and Δ*r* = 50 (◆) **(c**) Decorrelation length ${\lambda }_{{\rho }_{\hat{v}}}(\phi )$ from exponential fits to the large $\Delta r$ decay of the IAC functions.

The IAC curves calculated for different surface coverage values are shown in Fig. 8a. For the case of solitary twitchers corresponding to $\phi =0.004$, the principle contribution to ${\rho }_{\hat{v}}(\Delta r)$ is exponential decay, with a small dip and peak at small distances representing the stochastic angle chosen in the extension phase and the directed active motion of the retraction phase. In the low coverage regime ($\phi =0.19$), as *ϕ* increases the IAC curve shifts downward and also the decay becomes steeper. The shift reflects the same collisional dynamics as the short-time MSV decrease of ${V}^{2}(\phi )$ (Fig. 5c), while the increased decay reiterates the long-time MSD of $D(\phi )$ (Fig. 5d). If the coverage fraction is greater than ${\phi }^{\ast }$ ($\phi =0.57,0.76$), the slopes start to flatten out and the IAC ${\rho }_{\hat{v}}(\Delta r;\phi )$ shifts up in magnitude, implying that high coverages cage twitchers’ direction of motion as they travel large distances.

If one focuses on a subset of chosen travel distances $\Delta r=\{50,10\}$, the importance of ${\phi }^{\ast }$ is highlighted (Fig. 8b). For $\Delta r=50$, ${\rho }_{\hat{v}}(\Delta r;\phi )$ is non-monotonic, decreasing rapidly with *ϕ* to a minimum at ${\phi }^{\ast }$. For this large-distance limit, we characterize ${\rho }_{\hat{v}}(\Delta r;\phi )$ by fitting exponential correlation lengths ${\lambda }_{{\rho }_{\hat{v}}}(\phi )$ (Fig. 8c) to the tails of the curves in Fig. 8a. At low coverage fractions, ${\lambda }_{{\rho }_{\hat{v}}}(\phi )$ is largest due to unobstructed twitcher motion. The correlation length drops to a shallow minimum at $\phi \approx {\phi }^{\ast }$ with only a minor increase for larger coverage. The short distance ($\Delta r=10$) correlation indicates more complicated dynamics (Fig. 8b). The correlation still drops to a local minimum at $\phi \approx {\phi }^{\ast }$ but now the minimum is nearly 4.5 times more correlated than for $\Delta r=50$. The rise in ${\rho }_{\hat{v}}(10;\,\phi )$ above ${\phi }^{\ast }$ begins more suddenly and climbs to a local maximum around $\phi \approx 0.57$. At this local maximum, the IAC of a twitcher is nearly as large as for a solitary twitcher. At these coverages, twitchers form tightly packed clusters that promote alignment and cage the twitchers’ direction of motion, maintaining correlation. Thus, individual auto-correlation calculations can reveal the persistence of motion of individual *P. aeruginosa* or other motile microbes and by comparing the curves across *ϕ* values, the emergence of collective motion can be indirectly observed from increases in the IAC arising from interactions with neighboring twitchers.

### Long-range correlated motion

In order to directly examine these correlations between twitchers, we consider another correlation function: the radial pair auto-correlation (PAC) function given by

5 $${g}_{\hat{v}}(\Delta r;\phi )=\langle {\hat{v}}_{\gamma }(t)\cdot {\hat{v}}_{\eta }(t)\rangle .$$

This measure compares the direction of motion of the ${\gamma }^{{\rm{th}}}$ twitcher relative to its ${\eta }^{{\rm{th}}}$ neighbor that is a distance $\Delta r$ away at that instant. While the IAC given in Eq. 4 compares a twitcher to itself at different displacements and thus different times, the PAC given in Eq. 5 compares one twitcher to its neighbours at the same point in time. This is thus a direct measure of how the motion of a twitcher is correlated to that of its neighbours and allows us to explore the inference that tightly packed domains result in long-range correlated motion by caging twitchers and aligning their direction of motion. As for the IAC, values near +1 indicate high correlation while values near 0 indicate insignificant correlation.

**Figure 9:**
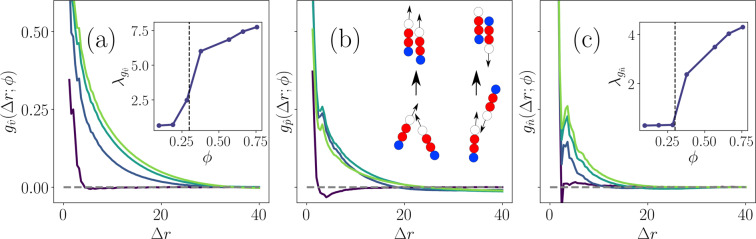
**Pair correlations between twitchers.** Radial pair auto -correlation (PAC) functions demonstrating local ordering for the same coverage fractions as in Fig. 3 $(\phi =\{0.19,0.38,0.57,0.76\})$. **(a)** PAC function of the direction of motion ${g}_{\hat{v}}(\Delta r;\phi )=\langle {\hat{v}}_{\gamma }(t)\cdot {\hat{v}}_{\eta }(t)\rangle$ for twitchers $\gamma$ and $\eta$ that are separated by $\Delta r$ at time $t$. (**Inset**) Exponential decorrelation length ${\lambda }_{{g}_{\hat{v}}}(\phi )$. **(b)** PAC function of polar orientation ${g}_{\hat{p}}(\Delta r;\phi )=\langle {\hat{p}}_{\gamma }(t)\cdot {\hat{p}}_{\eta }(t)\rangle$. (**Inset**) Schematic of steric alignment mechanisms for co-translating twitchers and passing twitchers. **(c)** PAC function of the director ${g}_{\hat{n}}(\Delta r;\phi )=\langle {\hat{n}}_{\gamma }(t)\cdot {\hat{n}}_{\eta }(t)\rangle$. (**Inset**) Exponential decorrelation length ${\lambda }_{{g}_{\hat{n}}}(\phi )$.

In dilute systems, the correlation of neighboring twitchers’ direction of motion drops quickly to zero (Fig. 9a) — only twitchers that are in direct contact (within $\Delta r < {L}_{{\rm{body}}}/2$) exhibit non-negligible correlations. However, there is a sudden jump in the long-range correlation as the coverage surpasses ${\phi }^{\ast }$. The principle contribution to ${g}_{\hat{v}}(\Delta r;\phi )$ is exponential decay and by fitting exponential correlation lengths ${\lambda }_{{g}_{\hat{v}}}(\phi )$ to the tail of the curves, we see the rapid rise and subsequent saturation of the correlation within the system. A closer examination reveals that there is a minor peak in ${g}_{\hat{v}}(\Delta r;\phi )$ for all *ϕ* found at small separations.

In Fig. 9a, we considered the PAC for the instantaneous direction of motion ${\hat{v}}_{\gamma }$. This does not necessarily align with twitchers’ polar orientation ${\hat{p}}_{\gamma }$, which describes the direction from twitcher’s tail to its head, nor twitchers’ nematic alignment ${\hat{n}}_{\gamma }$, which disregards differences between parallel/anti-parallel orientation (${\hat{n}}_{\gamma }=-{\hat{n}}_{\gamma }$), as defined in the Methods Section. An anti-correlation for $\phi  < {\phi }^{\ast }$ arises in the PAC function of polar orientation ${g}_{\hat{p}}(\Delta r;\phi )=\langle {\hat{p}}_{\gamma }(t)\cdot {\hat{p}}_{\eta }(t)\rangle$ (Fig. 9b). From ${g}_{\hat{p}}(\Delta r;\phi )$, we see that the $\phi =0.19$ curve crosses zero at $\Delta r=2.8$ and has a negative minimum at $\Delta r=4.0$. These features arise from pair collision events, which produce either:

1. Alignment, in which case the nematic interactions and polar motion cause persistent co-movement. Even if future pili adhesion events pull the heads apart, nematic interactions keep the pair aligned (Fig. 9b; left inset).
2. Anti-alignment, in which case the nematic interactions produce ephemeral anti-parallel configurations, since twitchers are free to move in uncorrelated directions once the twitchers pass one another (Fig. 9b; right inset).

The net result is that polar aligned twitchers have an effective short-range attraction and that twitchers in immediate contact tend to stay polar aligned. Since co-aligned twitchers effectively attract and anti-aligned do not, the range $\Delta r\approx 3-15$ exhibits an anti-correlation. This anti-correlated region has a minimum centered on the mean separation distance between twitchers ($\Delta r=4.0$ for $\phi =0.19$ in Fig. 9b). At higher *ϕ*, this is no longer the case, since spontaneous symmetry breaking is expected of active systems above the critical “flocking” transition^1,2^.

However, while ${g}_{\hat{v}}(\Delta r;\phi )$ increased for all *ϕ* (Fig. 9a), the $\phi =0.76$ curve for ${g}_{\hat{p}}(\Delta r;\phi )$ actually crosses down below the $\phi =0.38$ and $\phi =0.57$ curves for local $\Delta r$ (Fig. 9b). At these high coverages, the polar alignment mechanism described by Fig. 9b (inset) no longer holds since isolated pair collisions are rare. Anti-aligned pairs can no longer episodically pass one another because the majority of twitchers are surrounded on all sides by nearby neighbors (Fig. 2b). Thus, the coverage fraction in these dense regions nematically aligns the twitchers because of the bacilliform shape, overcoming collisional polar alignment.

This is revealed in the 2D pair -correlation function of nematic orientation ${g}_{\hat{n}}(\Delta r;\phi )=\langle 3({\hat{n}}_{\gamma }(t)\cdot {\hat{n}}_{\eta }(t)-2/3)\rangle$ (Fig. 9c). Unlike ${g}_{\hat{p}}(\Delta r;\phi )$, the magnitude of ${g}_{\hat{n}}(\Delta r;\phi )$ increases monotonically with *ϕ* at all $\Delta r$. The nematic PAC is very high at contact (small $\Delta r$) for all coverages, falls rapidly, then possesses a well-defined peak at intermediate separations $\Delta r\approx 4.0$; consistent to all three subplots. This peak corresponds to the length of a twitcher indicating that twitchers are often observed in locally smectic-ordered layers, as can be seen in Fig. 2b, for example. The locally correlated domains represent proto-rafts, regions of strong nematic ordering that are reminiscent of the “rafts” observed in dense communities *P. aeruginosa*^78,79^.

Fitting exponentials to the ${g}_{\hat{n}}(\Delta r;\phi )$ tails after the nematic raft peaks, we extrapolate an effective raft size parameter ${\lambda }_{{g}_{\hat{n}}}(\phi )$ (Fig. 7c; inset). These nematic proto-rafts have a size scale (Fig. 7c; inset) that is much smaller than the size of the dense regions, which can span the entire system at high *ϕ* (Fig. 2b). In this way, we see clearly the distinct transition from the dilute state with no clustering to a dense state with non-homogeneous polydomains of local nematic ordering that exhibit collective motion on scales comparable but larger than raft size. While local alignment on scales comparable to ${\lambda }_{{g}_{\hat{n}}}(\phi )$ generate the collective motion of rafts, ${\lambda }_{{g}_{\hat{v}}}(\phi ) > {\lambda }_{{g}_{\hat{n}}}(\phi )$ (Fig. 9; insets) since non-aligned neighbors can be entrained by the collective advection.

### Non-homogeneous ensemble structure

The nematic rafts represent ordered localities within larger dense regions. From Fig. 2b, it can be seen that at high total coverage fractions localized dense regions (liquid-like state with non-uniform polydomain nematic ordering) coexist with dilute regions (active gas-like state). To quantify the coexistence, we consider the distributions of local coverage fractions $\phi {\prime}$ by partitioning the system into 100 square sub-domains to calculate the probability distribution $P(\phi {\prime} ;\phi )$ of observing a local $\phi {\prime}$ given a certain global surface coverage *ϕ*.

**Figure 10:**
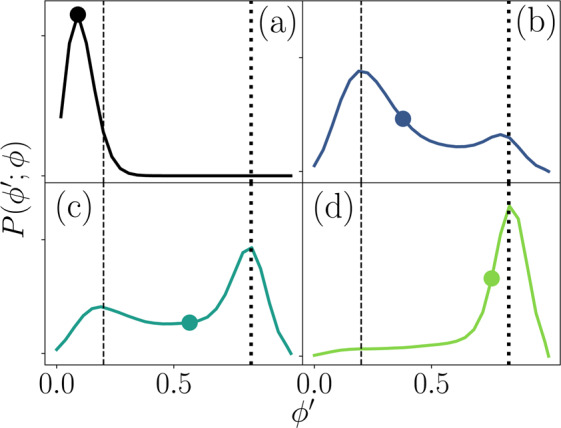
**Coexistence.** Probability distributions $P(\phi {\prime} ;\phi )$ of local coverage fractions $\phi {\prime}$ for different global coverage *ϕ*. Vertical lines denote the coexistence densities in the active gas-like phase ${\phi {\prime} }_{g}=0.2$ (dashed line) and the liquid-like dense phase ${\phi {\prime} }_{l}=0.85$ (dotted line). **(a)** $\phi =0.10$. **(b)** $\phi =0.38$. **(c)** $\phi =0.57$. **(d)** $\phi \mathrm{=0.76}$. Global *ϕ* is marked on each curve.

Below ${\phi }^{\ast }$, the distribution exhibits a single peak centered around $\phi {\prime} =\phi$, which is to say that the twitchers constitute a homogeneous gas-like active system (Figs. 10a and 2a). As the total coverage is raised, the primary peak shifts slightly to the right, as a secondary peak arises at a substantially larger coverage fraction (Fig. 10b). Above ${\phi }^{\ast }$, a dilute gas-like phase with coverage fraction ${\phi {\prime} }_{g}=0.2$ coexists with a liquid-like phase at ${\phi {\prime} }_{l}=0.85$. As the total *ϕ* is increased further, the fraction of twitchers that reside in the active-gas phase decreases, while the fraction in the active cluster increases (Figs. 10c and 2b). Eventually the active-gas phase all but disappears at the highest coverage fractions (Fig. 10d).

**Figure 11:**
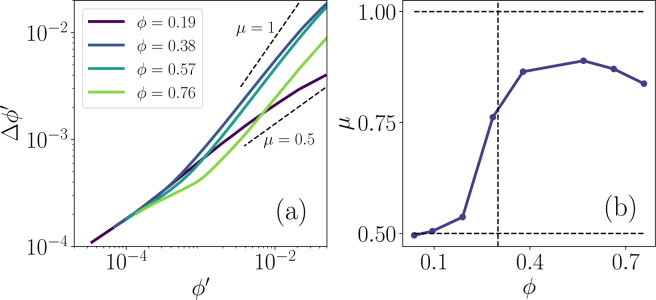
**Twitcher surface coverage fluctuations.** **(a)** Fluctuations of the local coverage $\Delta \phi {\rm{{\prime} }}$ as a function of the local instantaneous coverage $\phi {\prime}$ for various global coverage fractions *ϕ*. Reference scalings $\Delta \,\phi {\rm{{\prime} }}\sim \phi {{\rm{{\prime} }}}^{\mu }$ for $\mu =1/2$ and $1$ (dashed lines) are expected in the thermal-like and active-nematic limits respectively. **(b)** Power law exponent $\mu$ describing the scaling of the fluctuations with local coverage, as measured in the large $\phi {\prime}$ limit. Vertical dashed line denotes ϕ^*^.

However, even at these high coverages the impact of interspersed zones depleted of twitchers can be observed. Within the sub-domains of the system, we measure the fluctuations of the number of twitchers. That is, we measure the standard deviation $\Delta \phi {\prime} (\phi )={\langle {[\phi {\prime} (\overrightarrow{r},t;\phi )-\phi ]}^{2}\rangle }^{1/2}$ of the local coverage fraction for different subsection sizes (Fig. 11a). In the dilute limit, one expects $\Delta \phi {\prime} \sim {\phi }^{{\prime} \mu }$ with $\mu =1/2$ in accordance with the central limit theorem (CLT). However, as intrinsically far-from-equilibrium systems there should be no general expectations that density fluctuations of motile microbes obey the CLT. Indeed, in dense active nematic systems, giant number fluctuations (GNF) with $\mu =1$ are predicted^80^ and anomalous density fluctuations have been observed in simulations of self-propelled particles^81,82^ and experiments of driven granular matter^13,83^. Nevertheless, the scaling exponent $\mu$ may depend on microscopic details, such as shape and motility mode, or surface coverage, as we will now demonstrate. For $\phi  < {\phi }^{\ast }$, the fluctuations are thermal-like with $\mu =1/2$, as expected from CLT for the gas-like phase (Fig. 11b). However, $\mu$ is much closer to unity than $\mathrm{1/2}$ in the large *ϕ* limit (Fig. 11b). The transition from the CTL to GNF occurs rapidly about ${\phi }^{\ast }$. The increased fluctuations can be interpreted as a result of twitchers clustering together in dense actively flowing regions with polydomains of orientational ordering, while leaving depleted windows of low density between actively motile clusters. Together these combine to cause $\phi {\prime} (\overrightarrow{r},t;\phi )$ to swing from large to small values. In the small-$\phi {\prime}$/large-$\phi$ limit, the fluctuations are actually suppressed, rather than enhanced, because whole rafts of twitchers are caged within the liquid phase regions (Fig. 11). Similar giant number fluctuations have been, for example, reported in dense ensembles of swimming *B. subtilis*^74^.

## Discussion

Using coarse-grained and simplified simulations of bacilliforms with a stochastic motility cycle of rest, pilus extension and pilus retraction, we explored the collective behavior of twitchers as a function of surface covering. Although our study greatly simplifies twitcher-type bacteria by neglecting species-specific and biologically mediated complexities, we find cooperative action arising from physical mechanisms across all scales. By analyzing the displacement statistics of individual model twitchers within the ensemble, we found that the intermediate time shoulder in the mean squared displacement corresponding to twitchers in their resting period disappears with high coverage fraction, demonstrating that non-motile twitchers in their resting period are carried by the flow of their active neighbors. The MSD also showed that the short-time dynamics are slowed with the effective mean squared velocity decreasing monotonically with coverage. However, the long-time dynamics, as measured by relative diffusivity, are non-monotonic exhibiting an increase after a critical coverage fraction. This coverage fraction corresponds to the mean area per twitcher equaling the characteristic rotational area occupied by each bacilliform.

These conclusions more readily found by employing the non-Gaussian parameter, which provides additional information on the dynamics of the twitchers. The non-Gaussian parameter loses its negative plateau with higher coverage, which provides evidence of non-motile resting twitchers being displaced by the flow of active twitchers. Additionally, by separating the contributions to the average velocity due to twitchers in the retraction and rest phases, we find motile and non-motile twitchers are indistinguishable at sufficiently high coverage with their speeds converging to the mean. We can definitively conclude that not all cells must be motile in the collective clusters^38^. Furthermore, the increase of the short-time NGP with coverage fraction implies larger displacements than expected by a normal distribution. This is validated using the step size displacement distributions (van Hove self-correlation functions) which exhibit longer tails than a normal distribution indicating collective motion for higher coverage. While this does not imply twitchers exhibit bacterial turbulence^25,84,85^, it does reveal the early stages of collectivity in pre-biofilm twitching communities.

From the correlation functions, we see the microscopic arrangement of twitchers to form co-moving polar-aligned pairs in low coverage situations. However, the twitchers self-assemble into oriented local domains at high coverage, which form heterogeneous ordered polydomains within larger liquid-like regions, similar to bacterial rafts observed in bacterial colonies^78^. Biologically observed rafts generally move radially outward from the colony along the local alignment of the cells, which are in tight contact. As in our model proto-rafts, direction can vary and individuals within a raft may instantaneously move against the local flow but are advected with the group. An important distinction exists between the proto-rafts in our simulations and biological rafts in *P. aeruginosa* colonies—cells left behind biological rafts stretch and the continuity of the community breaks into small aggregates or even a network^79^. On the other hand, proto-rafts are free to simply move away from a larger cluster into a depleted region, forming a separate cluster since our model bacilliforms only interact via steric, excluded volume and not by signaling or other biological mechanisms. The transition from a purely dilute state with no clustering to a dense state with collective motion and non-homogeneous polydomains of local nematic ordering exhibits coexistence between the dilute and dense states. Such coexistence of separated phases appears to be a hallmark of self-propelled particles in general, not limited to twitching bacilliforms nor self-propelled rods, which has been studied theoretically in terms of motility-induced phase separation^86,87^, in simulations of active Brownian particles^18,19,88^, in self-propelled ballistic particles^23^, kinetic Monte Carlo^89^ and experimentally in systems of active spherical Janus colloids^24^. Similarly, our simulations quantify the giant number fluctuations and dynamic distributions of the coverage produced by twitching motility, which are likewise expected from active nematic systems^83,90^.

While our model is simplified compared to the biological complexity of *P. aeruginosa* and other bacteria that employ twitching as a motility strategy, microscopic details of swimming motility have previously been shown to result in qualitative changes to collective dynamics^26,91–93^ and swarming-mode motility of *P. aeruginosa*^94^. Our well defined microscopic model of the twitching mode motility cycle captures the essential microscopic details that differentiate biologically relevant twitching motility from a purely idealized toy model of self-propelled rods and demonstrates that twitching motility is sufficient to exhibit physically mediated collectivity, without requiring additional long-range complications, such as photosensing and quorum sensing^95^ or secretions^56^ or other forms of bacterial stigmergy^96^. Although lacking a clear signal in the first order statistics of mean squared displacement, the collectivity of twitchers above a critical coverage fraction can be directly quantified by higher order statistics, including the non-Gaussian parameter, decorrelation lengths and the scaling of the fluctuations with local coverage. Such physically mediated collective properties may bestow an advantage on pre-biofilm communities of twitchers by allowing regions of high coverage to potentially seed the formation of biofilms, while continuously preserving a subpopulation of disengaged individuals that are free to explore the surface with effectively isolated twitcher dynamics.

## Methods

### Simulation details

Our coarse-grained model of motile microbes treats individual twitchers as stiff rod-like bacilliforms discretized into four spheres, with a non-integrated dummy particle representing the action of bacterial pili (Fig. 1a). At all times $t$, each sphere $i$ of mass $m$ is located at a point ${\overrightarrow{x}}_{i}(t)$ and subject to thermal noise ${\overrightarrow{\xi }}_{i}(t)$, drag $-\zeta {\dot{\overrightarrow{x}}}_{i}(t)$, and conservative forces $-\overrightarrow{\nabla }V({\overrightarrow{x}}_{i},{\overrightarrow{x}}_{j\ne i})$ with other spheres $j\ne i$. Simulations are conducted using Langevin Dynamics^57,58^, evolving according to

6 $$m{\ddot{\overrightarrow{x}}}_{i}=-\zeta {\dot{\overrightarrow{x}}}_{i}-\overrightarrow{\nabla }V+\overrightarrow{\xi }.$$

Since bacteria are microscopic in scale and subject principally to biological sources of noise (see Section Motility Cycle), the temperature of the Gaussian noise is set to an arbitrarily low value of $T=2\times {10}^{-7}$ with the friction coefficient $\zeta =1$. Simulations use an integration step of $\Delta t=0.01$, such that $100$ integration steps constitute $\tau =1$ unit time step. Each simulation runs for ${10}^{8}$ integration steps in a 2-dimensional simulation box of size $100$ with periodic boundaries.

### Individual twitchers

To account for the excluded volume of twitchers, a shifted truncated Lennard-Jones (Weeks-Chandler-Anderson) potential acts between all integrated particle pairs $\{i,j\}$

7 $${V}_{{\rm{WCA}}}({r}_{ij})=(\begin{array}{cc}4\varepsilon \left[{\left(\frac{\sigma }{{r}_{ij}}\right)}^{12}-{\left(\frac{\sigma }{{r}_{ij}}\right)}^{6}\right]+\varepsilon , & {r}_{ij} < {r}_{{\rm{c}}}\\ \mathrm{0,} & {r}_{ij}\ge {r}_{{\rm{c}}},\end{array}$$

where ${r}_{{\rm{ij}}}=|{\overrightarrow{x}}_{i}-{\overrightarrow{x}}_{j}|$ is the separation between two particles. The particle size $\sigma =1$ sets the length scale and the energy $\varepsilon =1$ sets the energy scale. All quantities are expressed in terms of $\sigma$, $\varepsilon$, and $\tau$. The cutoff ${r}_{{\rm{c}}}={2}^{1/6}$ truncates the long-range potential and $\varepsilon$ shifts it.

Each twitcher body is composed of four spheres, bonded together by finitely extensible nonlinear elastic (FENE) potentials

8 $${V}_{{\rm{FENE}}}({r}_{ij})=-\frac{1}{2}{k}_{{\rm{F}}}{R}_{0}^{2}ln\left(1-{\left[\frac{{r}_{ij}}{{R}_{0}}\right]}^{2}\right),$$

where ${R}_{0}=1.5$ is the maximum extent of the bond and ${k}_{{\rm{F}}}=50$ is a spring constant. Harmonic bonds keep twitchers rigid with ${k}_{{\rm{H}}}=33$ in the potential

9 $${V}_{{\rm{HARM}}}({\theta }_{ijk})={k}_{{\rm{H}}}{({\theta }_{ijk}-{\theta }_{0})}^{2}\mathrm{/2,}$$

which keeps the angle ${\theta }_{ijk}$ between three sequential particles tightly centered around ${\theta }_{0}=\pi$. Each twitcher body has a size ${L}_{{\rm{body}}}=4$.

### Motility cycle

We model the process of twitching with a stochastic rule-based motility cycle and a single dummy pilus particle that actively pulls the twitcher forward. There are three phases in the model twitcher motility cycle (Fig. 1c):

1. The first is a **rest** phase, in which each twitcher does not undergo self-induced movement (Fig. 1c-1). In this rest phase, the pilus is not adhered to the surface and the twitcher only passively responds to external forces. A twitcher in the rest phase has a 10% chance per $\tau$ of stochastically transitioning out of this phase.
2. The second period is defined as the pili **extension**phase, in which each twitcher hypothetically extends then adheres its dummy pilus to the surface (Fig. 1c-2). This extension phase occurs over a set period of $10\tau$. As in the rest phase, the twitcher does not undergo self-induced movement during the extension process. At the end of this phase, the dummy pilus is instantly fixed to a point a distance ${L}_{0}=2.4$ away from the head particle, with an angle relative to the body stochastically drawn from a uniform distribution on $[-\pi /4,\pi \mathrm{/4}]$.
3. The third phase is the **retraction** phase, in which the twitcher is actively motile (Fig. 1c-3). During this phase, the twitcher’s head is pulled towards its fixed pilus adhesion point. A linear potential

10 $${V}_{{\rm{PILI}}}({r}_{\gamma })=-{k}_{{\rm{P}}}({r}_{\gamma }-{r}_{0})$$

where ${r}_{\gamma }(t)=|{\overrightarrow{x}}_{\gamma ,{\rm{H}}}-{\overrightarrow{x}}_{\gamma ,{\rm{P}}}|$ is the distance between the head at ${\overrightarrow{x}}_{\gamma ,{\rm{H}}}(t)$ and the pili adhesion point ${\overrightarrow{x}}_{\gamma ,{\rm{P}}}(t)$ of the ${\gamma }^{{\rm{th}}}$ twitcher, is used to model the average force exerted by multiple pili^59^. The spring constant ${k}_{{\rm{P}}}=1$ and ${r}_{0}=0.2$ is the strength of the pilus force and the cut off distance respectfully.

The retraction phase ends when one of three conditions are met:

i. The twitcher **reaches** its pilus adhesion point. This is achieved if ${r}_{\gamma }(t) < {L}_{{\rm{R}}}=0.2$ (Fig. 1c-3.i).
ii. The head of the twitcher is pushed too far from the adhesion point. This is said to occur if ${r}_{\gamma }(t) > {L}_{{\rm{S}}}=3$, causing the pilus adhesion to “**snap**” (Fig. 1c-3.ii).
iii. The twitcher adhesion is **exhausted**. Since an unobstructed twitcher takes roughly $10\tau$ to reach its pilus, ${t}_{{\rm{M}}}=70\tau$ is chosen as the maximum time a twitcher can try to reach its pilus adhesion point before the adhesion fails (Fig. 1c-3.iii).

Once any of these occur, the twitcher returns to the rest phase and the cycle repeats.

### Twitcher ensemble

Many twitchers are modeled simultaneously, explicitly interacting only through excluded-volume repulsion. We define the 2D surface coverage fraction

11 $$\phi ={A}_{{\rm{twitch}}}N/{A}_{{\rm{box}}}=3.7854\times {10}^{-4}N$$

where ${A}_{{\rm{box}}}={100}^{2}$ is the area of the box, ${A}_{{\rm{twitch}}}=3.7854$ is the area of one twitcher taken to be a rod of length 4 with circular caps, and $N$ is the number of twitchers in the simulation. This does not include the pili, which have no excluded volume. Our simulations span from the solitary twitcher system with $N=1$ ($\phi =4\times {10}^{-4}$) to $N=2000$ ($\phi =0.76$).

To analyze the individual and collective dynamics of the ensemble, we consider the state of each twitcher. The position ${\overrightarrow{x}}_{\gamma }(t)$ and average velocity ${\overrightarrow{v}}_{\gamma }(t)$ over 1 time unit $\tau$ of the ${\gamma }^{{\rm{th}}}$ twitcher are defined to be the center of mass values, ${\overrightarrow{x}}_{\gamma }(t)={\sum }_{i\in \gamma }{\overrightarrow{x}}_{i}(t)$ and ${\overrightarrow{v}}_{\gamma }(t)={\sum }_{i\in \gamma }{\dot{\overrightarrow{x}}}_{i}(t)$, with average speed ${v}_{\gamma }(t)=|{\overrightarrow{v}}_{\gamma }(t)|$. In addition to the ensemble and time averaged speed ${v}_{{\rm{m}}}\equiv \langle v\rangle$ of all twitchers, we consider the separate contributions due to twitchers in their self-motile retraction phase ${v}_{{\rm{a}}}\equiv {\langle v\rangle }_{{\rm{retr}}}$ and their non-motile resting/extending phases ${v}_{{\rm{r}}}\equiv {\langle v\rangle }_{{\rm{rest}}+{\rm{ext}}}$. The instantaneous direction of motion ${\hat{v}}_{\gamma }(t)={\overrightarrow{v}}_{\gamma }(t)/{v}_{\gamma }(t)$ of each twitcher does not necessarily align with its polar head/tail orientation ${\hat{p}}_{\gamma }(t)=({\overrightarrow{x}}_{\gamma ,{\rm{H}}}-{\overrightarrow{x}}_{\gamma ,{\rm{T}}})/|{\overrightarrow{x}}_{\gamma ,{\rm{H}}}-{\overrightarrow{x}}_{\gamma ,{\rm{T}}}|$, where ${\overrightarrow{x}}_{\gamma ,{\rm{T}}}$ is the tail position (Fig. 1a). In addition to polar ordering, we will consider the nematic alignment of the twitchers denoted by ${\hat{n}}_{\gamma }(t)\equiv -\,{\hat{n}}_{\gamma }(t)$, disregarding parallel/anti-parallel differences.

## Supplementary information

Supplementary Information.

Supplementary Information2.

Supplementary Information3.

Supplementary Information4.

Supplementary Information5.

Supplementary Information6.
